# Neural Substrates of Brand Love: An Activation Likelihood Estimation Meta-Analysis of Functional Neuroimaging Studies

**DOI:** 10.3389/fnins.2020.534671

**Published:** 2020-09-25

**Authors:** Shinya Watanuki, Hiroyuki Akama

**Affiliations:** ^1^Department of Marketing, Faculty of Commerce, University of Marketing and Distribution Sciences, Kobe, Japan; ^2^Institute of Liberal Arts/School of Life Science and Technology, Tokyo Institute of Technology, Tokyo, Japan

**Keywords:** brand love, brand relationship, brand loyalty, meta-analysis, neuroimaging

## Abstract

Brand love is a critical concept for building a relationship between brands and consumers because falling in love with a brand can lead to strong brand loyalty. Despite the importance of marketing strategies, however, the underlying neural mechanisms of brand love remain unclear. The present study used an activation likelihood estimation meta-analysis method to investigate the neural correlates of brand love and compared it with those of maternal and romantic love. In total, 47 experiments investigating brand, maternal, and romantic love were examined, and the neural systems involved for the three loves were compared and contrasted. Results revealed that the putamen and insula were commonly activated in the three loves. Moreover, activated brain regions in brand love were detected in the dorsal striatum. Activated regions for maternal love were detected in the cortical area and globus pallidus and were associated with pair bonds, empathy, and altruism. Finally, those for romantic love were detected in the hedonic, strong passionate, and intimate-related regions, such as the nucleus accumbens and ventral tegmental area. Thus, the common regions of brain activation between brand and romantic love were in the dorsal striatum. Meanwhile, no common activated regions were observed between brand and maternal love except for the regions shared among the three love types. Although brand love shared little with the two interpersonal (maternal and romantic) loves and relatively resembled aspects of romantic rather than maternal love, our results demonstrated that brand love may have intrinsically different dispositions from the two interpersonal loves.

## Introduction

In developing marketing strategies, it is crucial to establish emotional bonds between brand(s) and consumers. This relationship has been defined as brand love, brand attachment, and/or brand commitment (Fournier, 1998; Ahluwalia et al., 2000; Thomson et al., 2005; Carroll and Ahuvia, 2006; Park et al., 2006, 2009; Batra et al., 2012). These emotional relationships between consumers and brands can contribute to loyalty, corporate growth, and long-term profitability (Thomson et al., 2005; Carroll and Ahuvia, 2006; Albert et al., 2008; Khamitov et al., 2019). Consumers with strong emotional bonds to their favorite brands attribute positive traits to them and exhibit strong intentions in protecting their beloved brand if it is maligned (Park et al., 2008). These consumers prefer to maintain proximity with their beloved brand (Fournier, 1998; Thomson et al., 2005; Park et al., 2010) and are willing to pay a premium price (Hazan and Shaver, 1994). Along with progress in studies on this area, concepts on consumer-brand relationships have been proposed and analyzed via statistical studies incorporating factor analyses (Thomson et al., 2005; Carroll and Ahuvia, 2006; Ahuvia et al., 2008; Albert et al., 2009; Sarkar, 2011; Batra et al., 2012; Bagozzi et al., 2016). Khamitov et al. (2019) classified five constructs of consumer brand relationships based on: attachment, love, self-brand connection, identification, and trust. Thus, although consumer-brand relationships can have various aspects, they also have close similarities in emphasizing both emotional relationships and strong ties between brands and consumers beyond a simple like or dislike. Carroll and Ahuvia (2006) referred to these relationships as “brand love,” defined as “emotional and passionate feelings for any trademark.” Ahuvia (1992, 1993) considered the feeling of love toward various objects. After that, Ahuvia (2005a,b) introduced the idea of “brand love” as concepts related to loved brands, possessions, and consumption activities, although the term was not explicitly used until that time. Subsequently, Ahuvia et al. (2008) developed the brand love theory. Moreover, Batra et al. (2012) comprehensively demonstrated that brand love comprises several constructs (passion-driven behaviors; passionate desire to use/willingness to invest resources/things done in past, self-brand integration; desired self-identity/current self-identity/life meaning/attitude strength 1: frequent thoughts, positive emotional connection; intuitive fit/emotional attachment/positive affect, long-term relationship, anticipated separation distress, overall attitude valence, attitude strength 2: certainty/confidence). Lastovicka and Sirianni (2011) considered material possession love to be a love for an irreplaceable object, unlike brand love, and investigated the root of material possession love. The results of these studies indicated that the feeling of love for objects, including brand love, are complex and varied. Therefore, in the present article, we broadly interpret the term “brand love” as a feeling of love for objects (including brands, products, possessions, activities, place, etc.) formed by emotional relationships between objects and consumers in a consumption context. Thus, practitioners and researchers have conducted marketing studies to investigate the relationships between consumers and brands by directly and prudently applying interpersonal love relationships to consumer brand relationships.

In particular, love studies typified by Sternberg (1986) have strongly influenced investigations examining brand love. He proposed a triangular theory involving three components—intimacy, passion, and commitment—that are important for forming interpersonal love relationships. Shimp and Madden (1988) proposed eight types of consumer-object relationships by adopting Sternberg's love theory. Several studies have directly applied romantic love relationships to brand love (Whang et al., 2004; Sarkar, 2011; Sarkar et al., 2012). These studies tend to claim that both strong passion and strong emotive arousal are essential concepts in the relationship between consumers and brands, similar to romantic relationships between individuals. The concept of brand attachment is based on Bowlby's work (Bowlby, 1969). In attachment studies, the realm of attachment is defined as the relationship between parents and infants. However, marketing studies have extended this concept to the relationship between a person and an object. Park et al. (2008) defined brand attachment as “the strength of the cognitive and affective bond connecting the brand with the self.” Moreover, attachment style (anxiety or avoidance) to brands influences brand attitudes and can manifest as loyalty and preference (Swaminathan et al., 2009; Thomson et al., 2012; Mende et al., 2013). Based on the self-expansion theory (Aron and Aron, 1986), Ahuvia (1993, 2005a,b) has adapted it to a consumption context. Subsequently, Ahuvia et al. (2009) elaborated on Ahuvia's theory to propose the conditional integration theory. They classified the integration between self and loved objects (brands) into two types: the actual level of integration and the desired level of integration with the loved objects (brands). When a brand reaches high levels of integrations in both actual and desired types, consumers feel love for a brand. Reiman and Aron (2009) also included brand meanings in the self that can foster brand love. Escalas and Bettman (2003) proposed the concept of self-brand connections in terms of brand association.

Thus, applying interpersonal love relationships to brand love has some effectiveness; however, Batra et al. (2012) specifically criticized the idea that interpersonal love theories can be directly applied to brand love and mentioned the applicability of interpersonal love to brand love. It is important to assess the similarities and differences between interpersonal love and consumer-object relationships (Ahuvia, 1992, 1993, 2005a,b; Ahuvia et al., 2008, 2009; Albert et al., 2008, 2009, 2013; Albert and Valette-Florence, 2010; Batra et al., 2012; Albert and Merunka, 2013; Langner et al., 2015; Bagozzi et al., 2016). While the importance of self-identification was reported for both interpersonal love and brand love, altruistic concerns were not observed for brand love (Batra et al., 2012). Yoon et al. (2006) used neuroscience techniques to demonstrate that brand personality was not processed like human personality in the information system within the brains of consumers. Bagozzi et al. (2016) reported weaker intensity and passion for brand love than that for interpersonal love. Langner et al. (2015) used qualitative and quantitative approaches to verify the transferability of interpersonal love to brand love. Langner et al. (2016) observed no respondents with altruistic attitudes to brand love and that brand love was a less intense emotion than partner love. The authors proposed that brand love had a different emotional nature than that of interpersonal (romantic) love. In contrast, Carroll and Ahuvia (2006) and Albert and Valette-Florence (2010) confirmed that the concepts of interpersonal love were effective in measuring brand love.

Although significant findings on the applicability of interpersonal love have been reported, it remains unclear to what extent the properties overlap between interpersonal and brand love; in other words, whether the overlap is broad or narrow, and whether brand love shares intrinsic dispositions of interpersonal love. The present study considered maternal and romantic love as typical interpersonal and aimed to identify similarities and differences among the three love types (i.e., brand, maternal, and romantic) using a neuroscience approach. We performed a quantitative meta-analysis using the activation likelihood estimation (ALE) method (Turkeltaub et al., 2002) for neuroimaging studies to evaluate commonly- and uniquely-activated brain regions in the three love types across multiple studies. Meta-analyses are essential to determine the convergence of results across multiple independent studies.

## Materials and Methods

**Figure 1:**
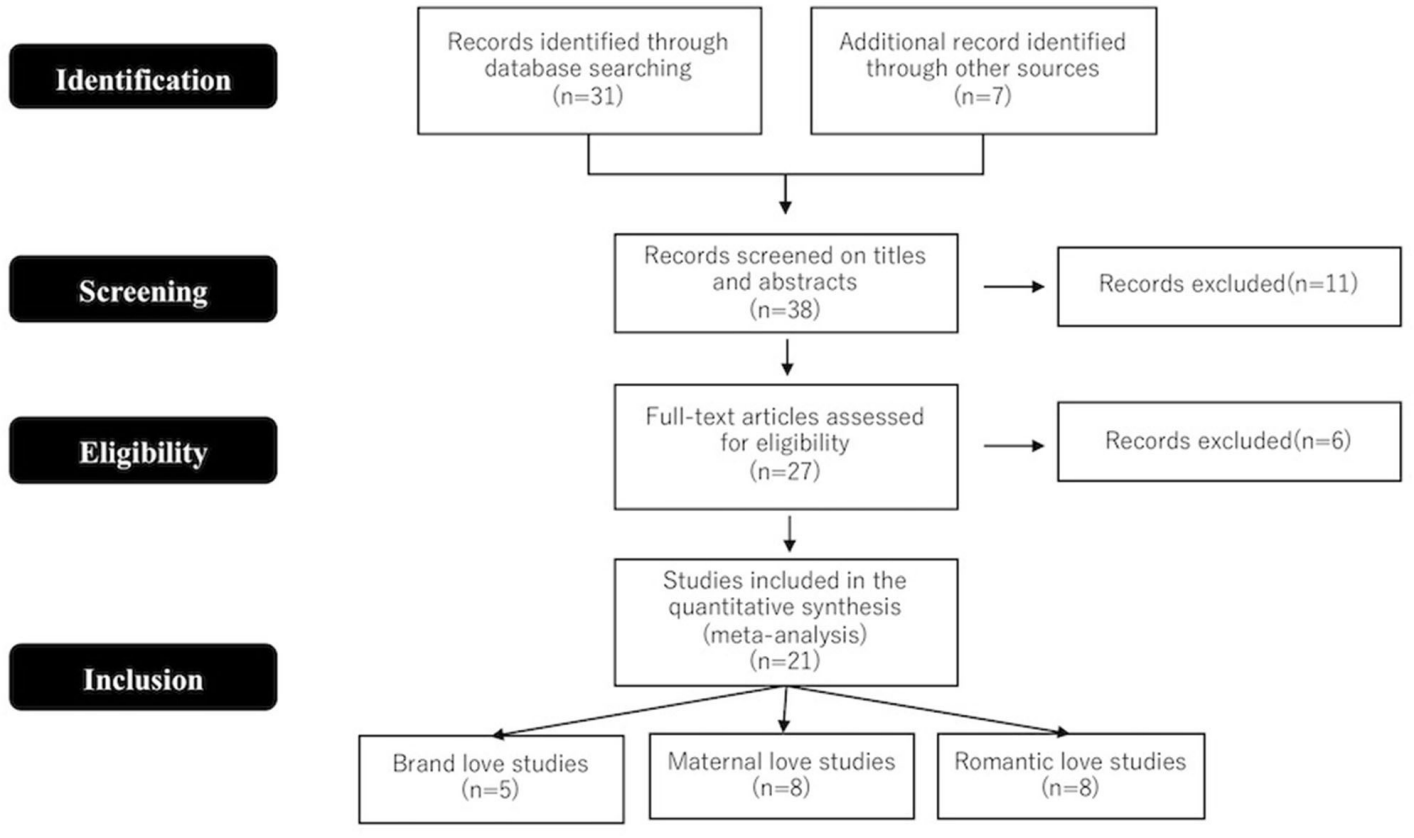
Prisma flow diagram.

Studies included in the present meta-analysis were selected by searching the NeuroSynth database [https://neurosynth.org/ (14,372 articles)] using the following terms: “romantic” for romantic love studies, “maternal or bonding” for maternal love studies, “brand or brand loyalty or brand love or brand attachment” for brand love studies. The literature search retrieved 31 articles. However, the literature search for studies in the field of brands and marketing was not solely restricted to the NeuroSynth database. As such, additional studies were retrieved by searching the Google Scholar database. Search terms included combinations of the following neuroimaging terms: “MRI,” “fMRI,” and “brain,” as well as marketing- and brand-related terms, such as “brand,” “brand loyalty,” “brand love,” and “brand attachment.” Seven articles were added using this search engine. After reading the titles and abstracts of these 38 articles, 11 were eliminated and the full texts of the remaining 27 were read and assessed based on the following inclusion criteria: peer-reviewed original research in an English language journal; healthy adult population; results reported Talairach or Montreal Neurological Institute (MNI) space; and reported brain activation images. For studies that reported results from more than one subject group, each group was treated separately in accordance with the approach of Turkeltaub et al. (2012). Since only one article (Reimann et al., 2011) reported images of brain activation without coordinates, they were estimated by comparing the images with coordinates in the MNI standardized brain image. Prisma flow diagram (Figure 1) gives details on the screening process.

Twenty-one studies were included in the present meta-analysis (Table 1). All Talairach coordinates were converted to MNI space using icbm2tal transform (www.brainmap.org) before the formal analysis (Lancaster et al., 2007). The ALE method, which is a coordinate-based quantitative meta-analysis method used to test for commonly-activated brain regions across different experiments, was used in the meta-analysis. In a comparison of alternative coordinate-based meta-analysis methods, such as kernel density analysis (Wager et al., 2004) and signed differential mapping (Radua and Mataix-Cols, 2009; Radua et al., 2010), ALE was found to produce results most comparable to image-based meta-analysis (Salimi-Khorshidi et al., 2009). In fact, ALE is the most preferred method for meta-analytical comparison of neuroimaging data because it is difficult to access full-brain activation images.

**Table 1:** Studies included in the meta-analysis.

	**Experiments**	**Subject**	**Foci**	**Experiment stimuli**
Brand love	Schaefer and Rotte (2007)	14	5	Brand logos
	Plassmann et al. (2007)	22	1	Closing
	Reimann et al. (2011)	12	2	Songs
	Schaefer et al. (2011)	16	1	Pictures of branded product
	Reimann et al. (2012)	16	6	Brand logos
Maternal love	Bartels and Zeki (2004)	19	30	Pictures
	Lenzi et al. (2008)	16	15	Pictures of facial expression
			7	
			15	
	Noriuchi et al. (2008)	13	14	Video clips
			24	
			43	
	Musser et al. (2012)	22	6	Cry sound
	Wan et al. (2014)	20	34	Video clips
			19	
		9	14	
		20	18	
	Atzil et al. (2017)	25	22	Video clips
	Ho and Swain (2017)	29	1	Cry sound
			3	
	Kluczniok et al. (2017)	27	23	Pictures of facial expressiion
			9	
			5	
Romantic love	Bartels and Zeki (2000)	17	13	Pictures of the faces
	Zeki and Romaya (2010)	24	8	Pictures of the faces
	Xu et al. (2011)	18	8	Pictures of the faces
			7	
			2	
	Acevedo et al. (2012)	17	30	Pictures of the faces
			26	
			9	
			24	
	Cooper et al. (2012)	39	4	Pictures of the faces
			3	
			15	
			5	
			5	
			1	
			2	
			2	
	Xu et al. (2012)	12	1	Pictures of the faces
		6	3	
	Cooper et al. (2013)	38	13	Pictures of the faces
			8	
			14	
	Ebisch et al. (2014)	24	1	Partner's hands/Ball
			2	

In ALE, all foci reported in the selected studies were modeled by creating three-dimensional Gaussian probability distributions centered at each focus (i.e., reported x, y, and z coordinates). These probabilities were then combined within and across experiments to produce a whole-brain map of ALE values for each voxel. This experimental ALE map compared a null hypothesis map representing the noise distribution. To determine the reliability of the ALE maps, a permutation procedure was applied to test the differentiation between true convergence of foci and random clustering (Eickhoff et al., 2009, 2012; Turkeltaub et al., 2012). ALE meta-analysis was performed using the GingerALE version 3.02 tool (http://www.brainmap.org/). The threshold was set at *p* < 0.001 uncorrected with a minimum cluster size of 100 mm^3^. There are a few reasons why we adopted the statistical criteria. First, GingerALE manual recommend the criteria. Secondly, uncorrected *p*-value < 0.001 is widely adopted as a statistical criterion (Carp, 2012; Eickhoff et al., 2012; Garrison et al., 2013). In addition, the reason why we adopted this cluster size is that the minimum cluster size of 100 mm^3^ can be thought of slightly more conservative size than recommended cluster size (Carp, 2012; Eklund et al., 2016). According to Carp (2012), 80 mm^3^ is the recommended cluster size. ALE maps were generated for each of the three loves types (i.e., brand, maternal, and romantic). In this study, all ALE coordinates were reported in MNI space. All activated brain images were exported as NIfTI files and overlaid onto a canonical anatomical T1 brain template in MNI space using Mango software version 4.1 (http://ric.uthscsa.edu/mango/). Conjunction analysis is needed to statistically investigate both similarity and discrepancy among the three loves, but it is difficult to perform conjunction analysis using GingerALE version 3.02. This is because sample size does not achieve the criterion recommended by GingerALE manual. However, we computed a voxel-wise hadamard product between the two result images of ALE to extract top 5% voxels, with the view of performing an approach similar to conjunction analysis but not using hypothesis test for evaluating significance. This analysis was performed using Matlab.

## Results

**Figure 2:**
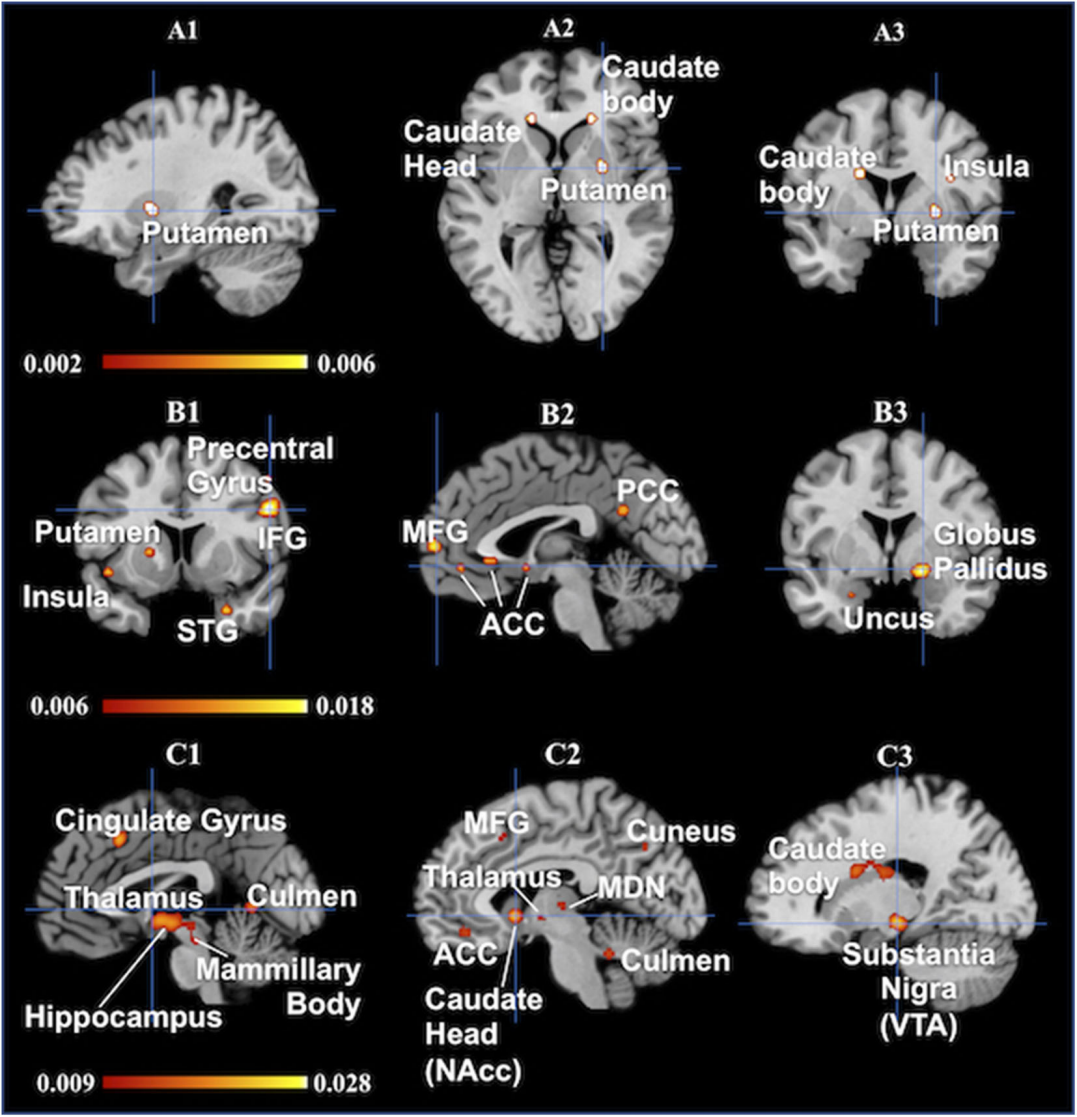
Results are from the ALE software for meta-analyses. All activations are significant at *p* < 0.001 uncorrected/**(A1–A3)**: Brand love, each of crosshairs is (28 0 0), **(A1)** sagital, **(A2)** Axial, **(A3)** coronal/**(B1–B3)**: Maternal love, **(B1)** sagital, Crosshair is (−2 59 −8), **(B2)** coronal, Crosshair is (−2 59 −8), **(B3)** coronal, Crosshair is (20 0 −10)/**(C1–C3)**: Romantic love, All views are sagital, **(C1)** Crosshairs is (−1 0 0), **(C2)** Crosshair is (−6 10 −4), **(C3)** Crosshair is (18 −20 −10). IFG, inferior frontal gyrus; STG, superior temporal gyrus; MFG, medial frontal gyrus; ACC, anterior cingulate cortex; PCC, posterior cingulate cortex; MDN, medial dorsal nucleus; NAcc, nucleus accumbens; VTA, ventral tegmental area.

Individual ALE maps were obtained for the three love types and ALE meta-analytical results are summarized in Tables 2, 3 and shown in Figure 2. Table 2 presents the coordinates and levels of the maximum ALE value. Table 3 is the brief version of Table 2. Results of the ALE analysis for the three love types are described below.

**Table 2:** Regional loci of brain activation by meta-analysis.

	**Brain regions**	**L/R**	**BA**	**Coordinate (MNI)**			**ALE**
				**X**	**Y**	**Z**	
**BRAND LOVE**							
	Caudate body	L	/	−8	14	4	0.0083
	Putamen	R	/	28	0	0	0.0081
	Putamen	L	/	−20	8	14	0.0079
	Insula	L	13	−34	8	16	0.0077
	Caudate body	L	/	−18	0	24	0.0077
	Caudate head	R	/	22	30	−2	0.0077
	Putamen	R	/	24	6	6	0.0077
	Caudate head	L	/	−16	30	0	0.0076
	Insula	R	13	38	2	20	0.0076
**MATERNAL LOVE**							
	Cuneus	R	18	6	−84	24	0.0234
	Hippocampus	L	/	−30	−30	−12	0.0230
	Inferior frontal gyrus	R	9	54	8	28	0.0213
	Insula	L	13	−44	12	−10	0.0195
	Middle temporal gyrus	R	37	58	−64	10	0.0181
	Precentral gyrus	L	6	−50	−4	4	0.0180
	Lateral globus pallidus	R	/	20	0	−10	0.0179
	Medial frontal gyrus	L	10	−2	62	4	0.0171
	Parahippocampal gyrus	R	28	26	−14	−20	0.0162
	Insula	L	13	−50	−34	22	0.0160
	Superior temporal gyrus	R	/	28	8	−32	0.0158
	Amygdala	L	/	−28	−4	−24	0.0155
	Putamen	L	/	−18	8	2	0.0150
	Anterior cingulate	L	24	−4	28	−4	0.0146
	Postcentral gyrus	R	1	66	−22	44	0.0142
	Anterior cingulate	L	25	−4	4	−10	0.0142
	Posterior cingulate	L	31	−2	−54	26	0.0141
	Superior temporal gyrus	L	39	−52	−54	8	0.0141
	Middle temporal gyrus	L	21	−58	−8	−22	0.0140
	Uncus	L	34	−22	4	−26	0.0139
	Middle temporal gyrus	R	21	60	−8	−22	0.0139
	Declive	L	/	−42	−60	−22	0.0139
	Anterior cingulate	L	32	0	44	−10	0.0139
	Middle frontal gyrus	R	6	52	10	48	0.0137
	Insula	R	13	48	−30	18	0.0137
	Inferior frontal gyrus	L	47	−28	22	−24	0.0136
	Superior temporal gyrus	L	38	−36	18	−32	0.0135
**ROMANTIC LOVE**							
	Cingulate gyrus	R	22	4	20	42	0.0370
	Hippocampus	R	/	34	−36	0	0.0337
	Putamen	L	/	−22	2	6	0.0243
	Substania nigra (VTA)	R	/	18	−20	−10	0.0241
	Caudate head (NAcc)	L	/	−6	10	−4	0.0239
	Mammillary body	L	/	2	−12	−8	0.0227
	Hippocampus	L	/	−34	−32	−4	0.0224
	Thalamus	L	/	0	−6	−6	0.0221
	Putamen	L	/	−20	2	18	0.0198
	Culmen	L	/	0	−60	2	0.0197
	Claustrum	R	/	40	12	−4	0.0196
	Insula	L	13	−44	6	−2	0.0183
	No gray matter found		/	2	−26	−20	0.0181
	Caudate head	R	/	10	4	−4	0.0181
	Caudate body	R	/	18	4	22	0.0178
	Caudate body	R	/	16	−12	22	0.0168
	Extra-nuclear	L	13	−38	8	−14	0.0164
	Hippocampus	L	/	−32	−46	2	0.0155
	Medial frontal gyrus	R	10	2	58	−10	0.0151
	Posterior cingulate	R	30	8	−52	16	0.0151
	Caudate tail	R	/	32	−44	12	0.0145
	Anterior cingulate	L	32	−6	40	−14	0.0144
	Lateral globus pallidus	L	/	−20	−2	−12	0.0142
	Anterior lobe	L	/	−6	−48	−28	0.0142
	Inferior frontal gyrus	R	47	36	28	−8	0.0139
	Cuneus	L	7	−8	−68	36	0.0138
	Medial globus pallidus	R	/	22	−6	−8	0.0136
	Medial dorsal nucleus	L	/	−6	−18	2	0.0131
	Culmen	R	/	6	−60	−20	0.0129
	Caudate head	R	/	8	12	−6	0.0129
	Superior occipital gyrus	L	19	−34	−72	36	0.0126
	Red nucleus	L	/	−2	−24	−10	0.0124
	Anterior cingulate	R	24	12	36	−10	0.0121

*BA, Brodmann area; L, left; R, right; ALE, activation likelihood estimation. The threshold was set at p 0 < 0.001 uncorrected for each ALE map*.

**Table 3:** Summary table for Table 2.

**Regions**		**Functions**	**Love types**		
			**Brand**	**Maternal**	**Romantic**
Basal ganglia	Putamen	Habitual behavior/reward/RL	Y	Y	Y
	Caudate	Valuation/reward/RL	Y		Y
	VTA	Motivation/reward/impulsive desire/RL			Y
	NAcc	Pleasure/reward/impulsive desire/RL			Y
	GP	Pair bond/empathy/altruism		Y	
Limbic	Amygdala	Emotional memory		Y	
	Hippocampus	Episodic memory		Y	Y
	Cingulate gyrus	Adjustment for emotion and cognition		Y	Y
Cortical regions	Insula	Subjective feelings/interoception/self-consciousness	Y	Y	Y
	Cuneus	Empathy/visual processing/working memory		Y	
	IFG	Empathy/executive function/working memory		Y	
	MTG	Empathy/object recognition/working memory		Y	

*VTA, Ventral Tegmental Area; NAcc, nucleus accumbens; GP, globus pallidus; RL, reinforcement learning; IFG, Inferior Frontal Gyrus; MTG, Middle Temporal Gyrus. “Y” means significant activated regions in each love types*.

### Brand Love

The greatest likelihood for brain activation in brand love was in the left caudate body, right putamen, and left putamen, followed by the left insula. Thus, ALE for brand love demonstrated a high convergence of activation mainly in the dorsal striatum, including the putamen and caudate. These regions are related to the reward system (Balleine et al., 2007), positive effect (Phan et al., 2002), reinforcement learning (Packard and Knowlton, 2002; Samejima et al., 2005), and the narrowing of preference target size in a choice set (Kim et al., 2014). Studies using positron emission tomography (PET) have reported that dopamine release is increased in the dorsal striatum during monetary reward tasks (Koepp et al., 1998; Zald et al., 2004). Thus, the caudate and putamen play crucial roles in the reward system of the brain. Moreover, it has been recently clarified that the caudate and putamen are engaged in different functional roles in reward learning. In the reward system, the caudate and anterior putamen play roles in reward expectation and the middle-posterior putamen coded values for habitual behaviors (Kawagoe et al., 1998; Gerardin et al., 2003; Haruno and Kawato, 2006; Balleine et al., 2007; Van Wouwe et al., 2012; Wunderlich et al., 2012; Lee et al., 2014). In addition, the caudate acts as an integrator in the brain valuation system that evaluates subjective values (Bartra et al., 2013; Audrin et al., 2017).

### Maternal Love

Brain regions with the most significant likelihood of activation associated with maternal love were the right cuneus, left hippocampus, and right inferior frontal gyrus (IFG), followed by the left insula. The other main activated regions were in the middle temporal gyrus (MTG), left precentral gyrus, right lateral GP, left medial frontal gyrus, right parahippocampal gyrus. Thus, in maternal love studies, the most activated brain areas were the region in the cortical area. The IFG and insula are regions related to social cognition such as cognitive empathy (Gallese et al., 2004; Chakrabarti et al., 2006; Riem et al., 2011). Both MTG pathways—the anterior cingulate/insula and the posterior cingulate/insula—are related to emotional empathy and inference from others' minds on inhibiting self (Jackson et al., 2006; Singer et al., 2006; Shibata and Inui, 2011). The combination of the precentral gyrus and insula is related to the theory of mind (Keysers and Gazzola, 2007). The cuneus are also the regions related to empathy and the theory of mind (Völlm et al., 2006). The function of the GP is related to the effects of oxytocin, prompted by strong altruism, pair bonding, and favoring derived from long-term relationships (Lim and Young, 2004; Lim et al., 2004; Acevedo et al., 2019).

### Romantic Love

Brain areas most associated with romantic love were the right cingulate gyrus, right hippocampus and left putamen, followed by the right substantia nigra, including the ventral tegmental area. The other main activated regions were in the left caudate head, including the nucleus accumbens, left mammillary body, left hippocampus, left thalamus, and left culmen. Thus, in romantic love studies, significantly elevated probabilities of activation in the basal ganglia area were more prominent in the midbrain. Moreover, the regions composed of Papez circuit (Papez, 1937) or related to the reward system (Phan et al., 2002) were activated. In particular, the ventral tegmental area and the nucleus accumbens are related to impulsive desire (Krämer and Gruber, 2015), pleasure (Olds and Milner, 1954), eliminating unpleasantness (Papoiu et al., 2013), financial reward (Thut et al., 1997; Delgado et al., 2000; Elliott et al., 2000; Knutson et al., 2000), and addiction (Gilpin and Koob, 2008).

### Conjunction Analysis

Results are shown in **Figure 4**. There were no overlapped regions between brand love and maternal love. On the conjunction between brand love and romantic love, we identified overlap voxels in the putamen and the caudate.

## Discussion

To the best of our knowledge, the present study was the first to review the neural correlates of brand love using ALE meta-analysis. Our meta-analysis aimed to identify the similarities and discrepancies in activated brain regions underlying brand love, maternal love, and romantic love. Although conjunction analysis was performed with the loose criterion mentioned above, we consider that results from conjunction analysis have effectiveness as a just reference. Therefore, we mainly investigated commonalities and differences among brain regions in the three love types by comparing brain-activated regions. With these considerations, we will discuss the uniqueness and characteristics of brand love compared with typical interpersonal loves, such as maternal and romantic love.

### Shared Brain-Activated Regions: Brand Love and the Typical Interpersonal Loves

**Figure 3:**
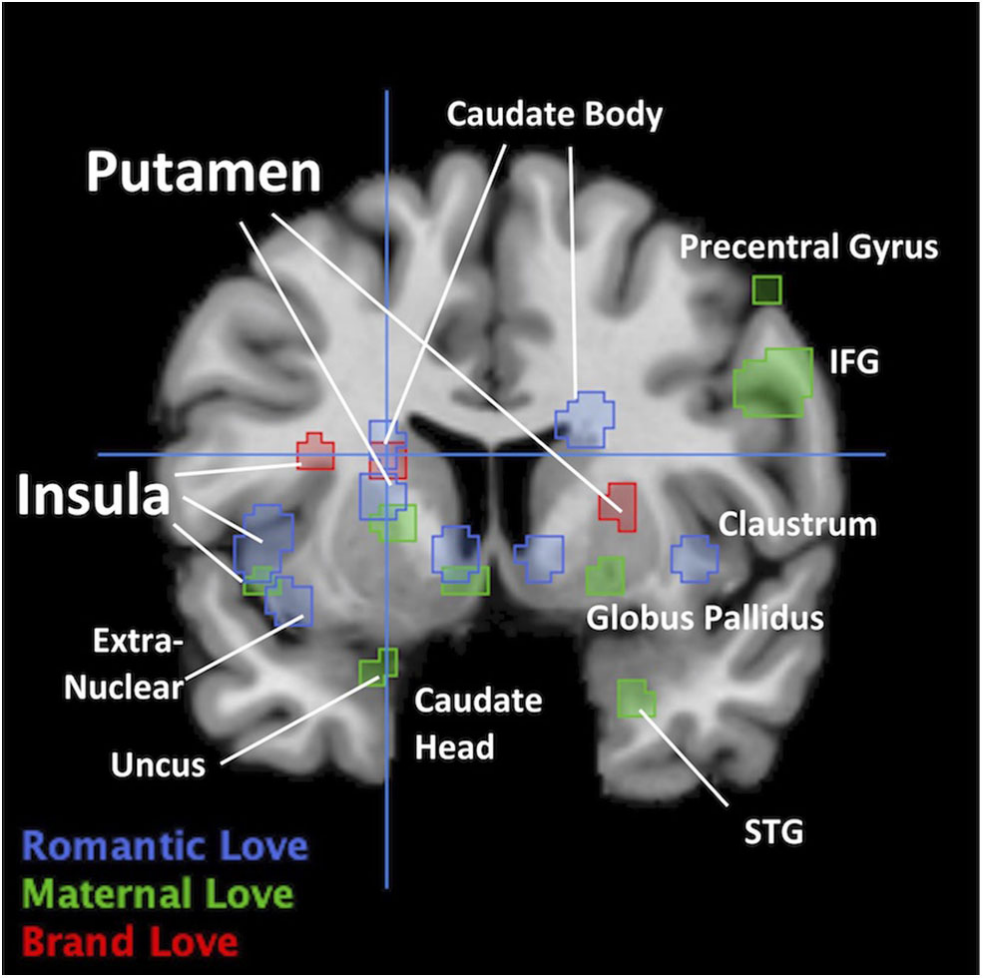
Overlapped regions across three loves in coronal view. Crosshair is (−20 7 15). IFG, Inferior Frontal Gyrus; STG, superior temporal gyrus.

Activation of the insula and putamen was commonly observed among the three love types (Tables 2, 3). In brand love, the activated region in the insula was the dorsal position compared with both maternal love and romantic love. However, the activated regions in maternal love and romantic love overlapped. In the putamen, activated regions among the three loves essentially overlapped (Figure 3).

Many studies have investigated the function of the insula. Investigations on the insula have demonstrated that the anterior insula plays an important role in interoception and subjective feelings (Caprara et al., 1985; Damasio et al., 2000; Iaria et al., 2008; Rudrauf et al., 2009; Terasawa et al., 2012, 2013; Zaki et al., 2012; Wang et al., 2019). Most studies have reported that brain-activated regions related to interoception and subjective feelings overlap (Caprara et al., 1985; Damasio et al., 2000; Iaria et al., 2008; Rudrauf et al., 2009; Terasawa et al., 2012, 2013; Zaki et al., 2012; Wang et al., 2019). In particular, Craig (2003) proposed the hypothesis that the most subjective feeling derived from interoception is engendered in the insula. Moreover, Craig and Craig (2009) further proposed that the insula is associated with self-consciousness and the accuracy of self-monitoring derived from the sense of body because Craig and Craig (2009) insisted that the “insula contained a somatotopic representation of subjective feelings of one's current movements as part of a representation of all feelings from body.” Thereby, our results indicate that the insula can be involved in self-related concepts according to marketing literature.

Previous studies have reported the importance of forming relevancy and integration between a brand and consumers' self-concept to build strong relationships and brand equity (Ahuvia, 1992, 1993, 2005a,b; Escalas and Bettman, 2003, 2015; Park et al., 2008; Ahuvia et al., 2009; Reiman and Aron, 2009; Batra et al., 2012). Ahuvia (1993, 2005a,b) and Ahuvia et al. (2009) suggested that the integration of the brand (the loved objects) into consumers' sense of identity brought out feelings of love for brands and objects in a consumption context. Escalas and Bettman (2015) argued that brands help consumers form their self-identity. According to their hypothesis, consumers can express who they want to be by possessing specific brands. These considerations are related to seeking the ideal self; moreover, achieving the ideal self produces higher self-esteem. Thus, self-identification and self-brand connections with brands play a crucial role in brand love. This result also indicates that activations in the insula are associated with “brand saliency.” The concept of “brand saliency” in the consumer's mind has been pointed out as an important idea in marketing strategy. Brand saliency involves keeping consumers aware and frequently reminded of a brand in order to occupy the largest share of the consumer's mind. Our results indicate that brain activation in the insula may be involved in brand saliency. More specifically, brand saliency is presumed to be a phenomenon derived from interoception. Moreover, it has been known that the insula plays a critical role in salience network and has been clarified that the salience network is involved in the detection of a self-referential information in the default mode network (Orliac et al., 2013). This implicates that brand saliency is a consumer's mind being involved in the salience network.

As mentioned above, many studies have reported that the putamen plays an important role in the reward network in the dorsal striatum. In particular, the anterior putamen and the caudate mediate goal-directed behaviors and deliberate decision making. In particular, the anterior putamen is involved in integrating and mediating information regarding the expectation of reward. On the other hand, it has been revealed that the posterior putamen is involved in coding value on habitual behavior such as repeated training (Balleine et al., 2007; Tricomi et al., 2009; Wunderlich et al., 2012; Dolan and Dayan, 2013). These roles in the putamen do not contradict one another. The anterior putamen is activated in the early stages, while activation of the posterior putamen increases in the later stages during the reward-based decision-making task (Gerardin et al., 2003; Lee et al., 2014). Therefore, the putamen works by maintaining a balance between the anterior and posterior parts according to circumstances and as necessary. However, symptoms, such as addiction, dependence, and compulsive-obsessive behaviors occur when this balance is disrupted (Van Wouwe et al., 2012; Sjoerds et al., 2013; Marsh et al., 2014). For example, alcohol-dependent patients exhibited stronger activation in the posterior putamen than healthy controls in an instrument task (Sjoerds et al., 2013). Moreover, the weak connectivity between the ventromedial prefrontal cortex and anterior putamen was observed in alcohol-dependent subjects when they acted habitually in the tasks compared to healthy controls. This means that the imbalance derived from impairing the executive control function in the frontal brain region causes an over-reliance on habit. In addition, it has been reported that reduced connectivity in the posterior insula-putamen is involved in cocaine addiction, relapse risk, and impulsivity (McHugh et al., 2013).

Our results demonstrated that the putamen is a shared brain region among the three love types and is an important substrate region for brand love. Many marketing studies have reported that frequency is one of the most crucial indicators of brand loyalty (Jacoby and Chestnut, 1978). Among academic marketing researchers and practitioners, it is widely known that consumers' purchasing behaviors are based on habit and have a strong tendency to persist (Pollak, 1970; Wood et al., 2002; Seetharaman, 2004; Quinn and Wood, 2005; Wood and Neal, 2009). It is difficult to make habitual consumers switch to other products they purchase habitually because a familiarity cue causes activation of associative responses (Wood and Neal, 2009; Riefer et al., 2017). Consumers have a tendency not to evaluate new products but existing products, since it is difficult and unfavorable for them to learn new usage behaviors (Murray and Häubl, 2007). Therefore, habitual behaviors without uncertainty generate fluency. Furthermore, fluency without cognitive load leads to familiarity. Ultimately, familiarity leads to positive feelings toward making decisions about choosing a brand (Wood and Rünger, 2016).

Based on the considerations above, love, including brand love, is based on certain repetitive relationships and is strongly involved in self-related emotions derived from interoception. In addition, both a motivation for goal-directed action and a habituation dependent on pleasantness derived from achieving its goals can reinforce relationships between subjects and objects.

In other words, brand love can be thought of as an information processing system based on the reinforcement learning system and related to fast and heuristic thinking involving subjective feelings in the context of dual process theories (Evans, 2008; Stanovich, 2008; Kahneman, 2011).

### Different Brain-Activated Regions: Brand Love and Typical Interpersonal Loves

Characteristic brain-activated regions in maternal love compared with brand love include the cortical regions and the GP. As mentioned, cortical regions activated in maternal love are related to social cognition such as empathy, theory of mind, and caregiving (Gallese et al., 2004; Chakrabarti et al., 2006; Jackson et al., 2006; Singer et al., 2006; Keysers and Gazzola, 2007; Riem et al., 2011; Shibata and Inui, 2011). In addition, the GP is involved in pair bonding and altruism, which are kinds of social cognition (Lim and Young, 2004; Lim et al., 2004; Acevedo et al., 2019). Some studies on brand love also have considered empathy as a crucial element in brand love (Fournier and Alvarez, 2012; Kervyn et al., 2012). However, in our meta-analysis, these social cognition-related brain regions were not activated in brand love; as such, we assume that brand love has a very weak disposition toward social cognitive aspects. In particular, the deactivation in the GP showed that brand love may not have as strong an aspect of social cognition as those observed for pair bonds and altruism. Batra et al. (2012) suggested that altruism is not a part of brand love despite containing altruistic concerns that are crucial elements in interpersonal love. Langner et al. (2015) also made the same point. Moreover, Albert et al. (2013) showed that altruism had no effects on building brand love. Thus, while our results support these findings, oxytocin reportedly enhances customer-brand relationships (Fürst et al., 2015). Oxytocin is a neuropeptide that is known to modulate the formation of social relationships, such as pair bonds and emotional empathy, and behavior (Lim and Young, 2004; Lim et al., 2004; Riem et al., 2011; Geng et al., 2018; Yao et al., 2018; Acevedo et al., 2019; Kruppa et al., 2019; Xu et al., 2019). Lastovicka and Anderson (2014) reported that consumer-object relationships such as playing with and nurturing material objects may increase oxytocin levels, implying that brand love could have social cognitive dispositions with activation in brain regions associated with oxytocin such as the GP even if consumer-brand relationships (a consumer-object relationship), are one-way, unlike interpersonal love. Previous brain activation studies investigating brand love could not detect oxytocin-related regions using assessment methods such as the current respondent assessment scales. However, it is possible that future novel experimental designs and procedures could reveal activation in oxytocin-related brain regions represented by pair bonds and altruistic dispositions.

**Figure 4:**
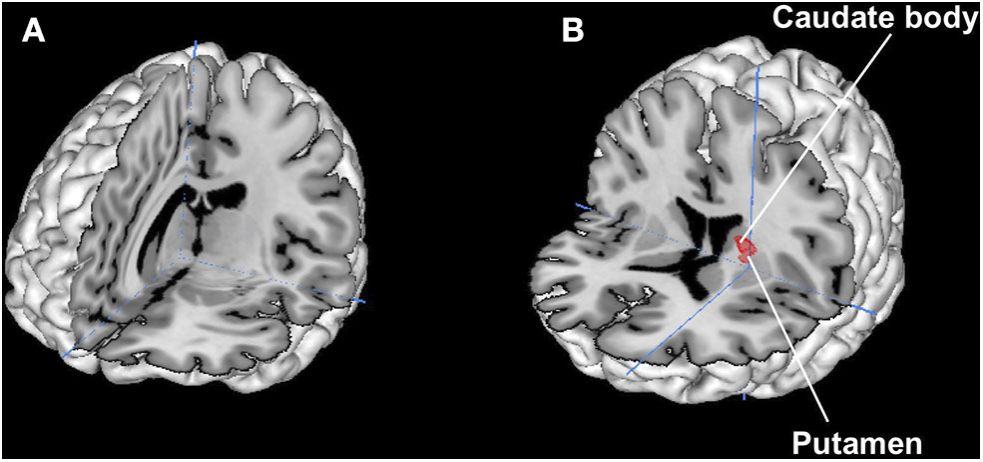
Results of conjunction analysis. **(A)** Brand love and Maternal love. No overlapped regions, crosshair is (7 −18 10)/**(B)**: Brand love and Romantic love. Overlapped regions are the putamen (−20 6 16), (−21 5 15), and the caudate body (−19 7 16), crosshair is (−21 1 4).

In contrast, romantic love shares more common brain-activated regions with brand love than maternal love, especially in the dorsal striatum (the putamen and the caudate, see Tables 2, 3, Figure 4). Other brain activation in romantic love characteristically converged regions related to thalamo-cortico-thalamic circuits consisting of the cortical regions, thalamus, and ventral striatum. The brain regions activated in romantic love are involved with impulsive desire and addiction (Gilpin and Koob, 2008; Krämer and Gruber, 2015). However, activation of these brain regions was not observed in brand love. Therefore, both romantic and brand love are involved in attitudes toward certain rewards related to goal-oriented and habitual behaviors (Kawagoe et al., 1998; Gerardin et al., 2003; Haruno and Kawato, 2006; Balleine et al., 2007; Van Wouwe et al., 2012; Wunderlich et al., 2012; Lee et al., 2014). Romantic love, however, showed dispositions toward more impulsive, intimate, and passionate relationships compared to brand love. The marketing literature stresses the importance of building impulsive, intimate, and passionate relationships between brands and consumers (Shimp and Madden, 1988; Fournier, 1998, 2014; Keller, 2001; Thomson et al., 2005; Carroll and Ahuvia, 2006; Park et al., 2008; Albert et al., 2009; Albert and Valette-Florence, 2010; Lastovicka and Sirianni, 2011; Sarkar, 2011; Batra et al., 2012; Sarkar et al., 2012; Cui et al., 2018; Mrad, 2018). Thus, while these strong emotions are important elements for brand love, we did not observe activation of brain regions related to impulsive, intimate, and passionate relationships in brand love in the present study. This finding suggests that brand love is not as strong an impulsive emotion as romantic love or as asserted in other studies on brand love. From a consumption context, consumers do not expect a strong reciprocal desire and passion despite being loyal and committed customers who might expect rewards from their loved brand, although these intensive emotions have been thought of as crucial concepts for romantic love (Ahuvia, 2005b; Albert et al., 2013). Ahuvia (2005b) demonstrated that several concepts of strong emotive arousal such as sexual arousal in interpersonal love were irrelevant in a consumption context. Bagozzi et al. (2016) demonstrated that most consumers did not experience intense emotion for brands. Langner et al. (2015) also verified that brand love is a weaker intensive emotion than that for loved persons based on results of the self-assessment manikin (SAM) arousal scale and skin conductance. Therefore, our results support these considerations (Ahuvia, 2005b; Albert et al., 2013; Langner et al., 2015; Bagozzi et al., 2016).

The results of our comparisons between brand love and two interpersonal loves (maternal and romantic love) demonstrated different dispositions for brand love from the interpersonal loves, although these loves also showed similarities. These findings suggest that the feelings of love for objects depend on the context of the relationship (Ahuvia, 1992, 2005a; Ahuvia et al., 2008; Lastovicka and Sirianni, 2011; Batra et al., 2012; Lastovicka and Anderson, 2014). In addition, Lastovicka and Sirianni (2011) proposed that material possession love was rooted in deficits in interpersonal relationships, although this is a non-interpersonal love. Thus, our results indicate that theories of interpersonal love should be prudently applied to brand love as the feelings of love for an object are complex concepts formed under the influence of various relationships (Ahuvia, 1992, 1993, 2005a,b; Albert et al., 2008, 2009, 2013; Ahuvia et al., 2009; Albert and Valette-Florence, 2010; Batra et al., 2012; Albert and Merunka, 2013; Langner et al., 2015; Bagozzi et al., 2016). The results of our study provide marketers useful views regarding the influence of love relationship styles on the formation of feelings of love for objects.

### Limitations and Future Research

Our study also had several limitations, the most important of which was that we had no choice but to perform ALE meta-analysis for brand love using a smaller sample size (*n* = 5 experiments) due to the scarcity of brain activation studies addressing brand love. A sample size of least 10–15 experiments are required to reduce the likelihood that meta-analytic results are unduly affected by a single experiment (Eickhoff and Bzdok, 2013). Due to this limitation, we could not conduct an appropriate conjunction analysis among the three loves by using a statistical approach. We would like to address the same issue after accumulating more brain imaging studies on consumer brand relationships. Moreover, we think considerations were needed in terms of demographic attributes such as age and sex, and psychographic, various product categories, the kinds of loved objects, and more detailed context regarding the relationships and their interactions since there can be various ways of thinking about love concepts depending on these attributes.

The results of our study contribute to the literature because, to our knowledge, it was the first ALE meta-analysis to reveal shared and different brain-activated regions between brand love and interpersonal love relationships, such as maternal love and romantic love. This study focused on comparisons between brand love and two interpersonal loves (maternal and romantic); however, as mentioned above, the feelings of love for objects are complicated and varied concepts that depend on objects that are loved and the context of the relationships. Therefore, further research is needed to address these limitations.

## Conclusion: What Is Brand Love?

Our major findings were that (1) brand love is typically less passionate and intense than interpersonal love (especially romantic love) (Ahuvia, 2005b; Albert et al., 2013; Langner et al., 2015; Bagozzi et al., 2016); (2) altruistic elements were not observed in brand love, although these are crucial elements in interpersonal love (Batra et al., 2012; Albert et al., 2013; Langner et al., 2015); (3) the core of brand love involves incorporating the loved object into the self (Ahuvia, 1992, 1993, 2005a,b; Escalas and Bettman, 2003, 2015; Thomson et al., 2005; Park et al., 2006, 2008, 2009; Ahuvia et al., 2008; Albert et al., 2008, 2009, 2013; Reiman and Aron, 2009; Lastovicka and Sirianni, 2011; Batra et al., 2012; Albert and Merunka, 2013; Bagozzi et al., 2016; Khamitov et al., 2019). Our findings support those of previous studies on brand love. Therefore, we concluded that brand love differed from the two interpersonal loves (maternal and romantic love).

## Data Availability Statement

The datasets generated for this study are available on request to the corresponding author.

## Author Contributions

SW: conceptualization, data curation, investigation, and writing. SW and HA: analysis, methodology, and supervision. All authors contributed to the article and approved the submitted version.

## Conflict of Interest

The authors declare that the research was conducted in the absence of any commercial or financial relationships that could be construed as a potential conflict of interest.

## Abbreviations

ALE: Activation Likelihood Estimation
GP: Globus Pallidus
MNI: Montreal Neurological Institute
PET: Positron Emission Tomography
IFG: Inferior Frontal Gyrus
MTG: Middle Temporal Gyrus.
